# Intimate partner violence and HIV infection among women: a systematic review and meta-analysis

**DOI:** 10.7448/IAS.17.1.18845

**Published:** 2014-02-13

**Authors:** Ying Li, Caitlin M Marshall, Hilary C Rees, Annabelle Nunez, Echezona E Ezeanolue, John E Ehiri

**Affiliations:** 1 Department of Social Medicine & Health Service Management, Third Military Medical University, Chongqing, China; 2 Division of Health Promotion Sciences/Global Health Institute, Mel & Enid Zuckerman College of Public Health, University of Arizona, Tucson, AZ, USA; 3 Department of Pediatrics, University of Nevada School of Medicine, Las Vegas, NV, USA

**Keywords:** intimate partner violence, women's health, systematic review, meta-analysis, gender-based violence, HIV/AIDS

## Abstract

**Introduction:**

To assess evidence of an association between intimate partner violence (IPV) and HIV infection among women.

**Methods:**

Medline/PubMed, Embase, Web of Science, EBSCO, Ovid, Cochrane HIV/AIDS Group's Specialized Register and Cochrane Central Register of Controlled Trials were searched up to 20 May 2013 to identify studies that examined the association between IPV and HIV infection in women. We included studies on women aged ≥15 years, in any form of sexually intimate relationship with a male partner.

**Results:**

Twenty-eight studies [(19 cross-sectional, 5 cohorts and 4 case-control studies) involving 331,468 individuals in 16 countries – the US (eight studies), South Africa (four studies), East Africa (10 studies), India (three studies), Brazil (one study) and multiple low-income countries (two studies)] were included. Results were pooled using RevMan 5.0. To moderate effect estimates, we analyzed all data using the random effects model, irrespective of heterogeneity level. Pooled results of cohort studies indicated that physical IPV [pooled RR (95% CI): 1.22 (1.01, 1.46)] and any type of IPV [pooled RR (95% CI): 1.28 (1.00, 1.64)] were significantly associated with HIV infection among women. Results of cross-sectional studies demonstrated significant associations of physical IPV with HIV infection among women [pooled OR (95% CI): 1.44 (1.10, 1.87)]. Similarly, results of cross-sectional studies indicated that combination of physical and sexual IPV [pooled OR (95% CI): 2.00 (1.24, 3.22) and any type of IPV [pooled OR (95% CI): 1.41 (1.16, 1.73)] were significantly associated with HIV infection among women.

**Conclusions:**

Available evidence suggests a moderate statistically significant association between IPV and HIV infection among women. To further elucidate the strength of the association between IPV and HIV infection among women, there is a need for high-quality follow-up studies conducted in different geographical regions of the world, and among individuals of diverse racial/cultural backgrounds and varying levels of HIV risks.

## Introduction

Intimate partner violence (IPV) is an important global health issue [1]. The 2010 Global Burden of Disease Study ranks IPV as 5th in years of life lost as a result of disability for women [2]. IPV may take various forms, including physical, sexual and psychological [1, 3]. Globally, it is estimated that one in four women experiences violence from an intimate partner in her lifetime, making IPV the commonest form of violence against women [4, 5]. Although both men and women experience IPV, women experience significantly higher rates than men, and suffer more injury or death as a result. While some of the biological explanations of the association between IPV and HIV are speculative, several empirical studies have demonstrated strong plausibility for this relationship. For example, victims of IPV may suffer negative health impact, ranging from fatal health outcomes, such as suicide, homicide, maternal mortality and AIDS-related mortality, to non-fatal health outcomes, such as injury, substance abuse, chronic pain and mental disorders [6–8]. The reproductive health of victims can be affected, with compelling research evidence for an association between IPV and unsafe sexual practices, unintended pregnancy and unsafe abortion [9–11]. IPV during pregnancy has been shown to be associated with adverse birth outcomes, including low birth weight, preterm delivery and small for gestational age [12, 13]. A landmark multi-country study commissioned by the World Health Organization (WHO) to assess the global burden of IPV between 1998 and 2004 [6, 14] confirmed a high burden of lifetime IPV among ever-partnered women, from 15% in Japan to 71% in Ethiopia [1, 14].

IPV and HIV are hypothesized to have critical intersections, and women's vulnerability to HIV may be influenced by violence caused by culturally accepted gender inequalities [15]. Dunkle and Decker [16] have provided a succinct expose on the possible intersection of IPV with HIV infection. For example, direct transmission of HIV can result from forced sexual intercourse with a HIV-positive male intimate partner. The biological risk of transmission in a violent sexual encounter may be higher for anal sex followed by vaginal and oral sex. Risk of HIV transmission may also increase with the degree of trauma, vaginal lacerations and abrasions that occur when force is used [15, 16]. Where sexual violence occurs in girls and young women, the risk of transmission is also likely to be higher because girls’ vaginal tracts are immature and tear easily during sexual intercourse [17, 18]. Evidence also shows that HIV transmission risk is higher in the presence of other STIs and when exposed to sexual secretions and/or blood [19–21].

With regard to indirect transmission of HIV as a result of IPV, some researchers have demonstrated a link between experience of IPV and increased sexual risk behaviours, including substance abuse, having multiple sexual partners or engagement in transactional sex [16]. Studies conducted in South Africa [19, 20] showed that women who experienced IPV were two to three times more likely to engage in transactional sex than women who did not [19]. Also, women who experienced IPV were six times more likely to use condoms inconsistently than those who did not experience IPV [20].

Although the relationship between IPV and HIV infection in women is generally accepted, findings of available studies are conflicting [16, 21, 22]. Unfortunately, prior reviews [23, 24] focused on the relationship between IPV and HIV infection among high-risk women (sex workers, alcohol abusers and women who experienced coerced/forced first sexual intercourse as young girls) who may not represent the general population of women globally. Thus, there is a need to explore the IPV-HIV infection hypothesis among the general population of women globally since the biological and contextual factors that underpin are often magnified in intensity among high-risk groups [16]. The objective of this review was to assess evidence of an association between IPV and HIV infection among the general population of women globally.

## Methods

### Case definition

We used the WHO [15] definition of IPV which includes *physical violence* (slaps, punches, kicks, assaults with a weapon, homicide), *sexual violence* (rape, coercion and abuse, use of physical force, verbal threats and harassment to have sex, unwanted touching or physical advances, forced participation in pornography or other degrading acts that often persist over time), *psychological violence* (belittling the woman, preventing her from seeing family and friends, intimidation, withholding of resources, preventing her from working or confiscating her earnings), and *any violence* (a combination of physical, sexual and psychological violence perpetrated by a male individual against a female intimate partner).

Inclusion/exclusion criteria: [1] Type of studies: Case-control studies, randomized controlled studies, cross-sectional studies and cohort studies that investigated the association between IPV (physical, sexual, psychological or their combination) and HIV infection among women. [2] Study population: Females aged 15 years and over, who were in any form of sexually intimate relationship (married, co-habiting, dating) with a male partner. This excluded childhood violence, violence by other family members/relatives and violence out of the home by strangers. [3] Outcome measures: HIV infection where HIV status was confirmed by laboratory test. We included studies that assessed the relationship between IPV and sexually transmitted infections (STIs) if they specifically collected and analyzed data for HIV/AIDS as a type of STI. We excluded studies that focused on high-risk women and special populations, including female commercial sex workers, alcohol and substance abusers, persons with severe mental illness and prisoners. Studies that assessed the relationship between abuse in childhood and HIV/AIDS incidence/prevalence in adulthood were excluded. Application of the inclusion and exclusion criteria to identified studies was done by two reviewers (YL and JE).

### Search strategy and selection criteria

To identify eligible studies published between January 1980 and 20 May 2013, we searched Medline/PubMed, Embase, Web of Science, EBSCO (PsycINFO and CINAHL), Ovid, the Cochrane HIV/AIDS Group's Specialized Register and the Cochrane Central Register of Controlled Trials. We sought unpublished data from the grey literature through Google and Google Scholar. We hand-searched reference lists of identified articles. The search was not restricted by publication status or language. The search terms included “HIV”[Mesh]) AND “Violence”[Mesh]) AND “HIV”[Mesh]/“HIV infection”[Mesh]; “Spouse”[Mesh] AND “Violence”[Mesh] AND “HIV”[Mesh] “HIV infection”[Mesh]; “Sexual Behavior”[Mesh] AND “Violence”[Mesh] AND “HIV”[Mesh]; “HIV infection”[Mesh]; “Spousal Violence” AND “HIV”; “IPV” AND “HIV/AIDS”; “Gender-Based Violence” AND “HIV/AIDS”; “Sexual Violence” AND “HIV/AIDS”; “Physical Violence” AND “HIV/AIDS”; “Wife Beating” AND “HIV”; “Wife Battering” AND “HIV”; “Domestic Abuse” AND “HIV”; “Domestic Violence” AND “HIV”; “Dating Violence” AND “HIV”. Three reviewers (YL, AN and JE) conducted the literature searches.

### Study selection

Two reviewers (YL and JE) independently screened titles and abstracts of identified studies to assess their eligibility for inclusion in the review. Where there were uncertainties regarding eligibility of studies, all reviewers participated in the decision about inclusion.

### Study quality assessment

We assessed the quality of case-control and cohort studies using the Newcastle–Ottawa Scale [25]. For case-control studies, we assessed the adequacy of case and control definition, representativeness of the cases, whether controls were derived from the same population as cases, comparability of cases and controls on the basis of design and analyses, ascertainment of exposure and non-response rates. For cohort studies, we assessed the representativeness of the exposed cohort in the study setting, the selection of non-exposed cohort, ascertainment of exposure, demonstration that outcome of interest was not present at start of the study, comparability of cohorts on the basis of design and analyses, outcome assessment and adequacy of follow-up [25]. After reviewing the quality of each included study on the basis of these criteria, we assigned a composite quality score of 0–9. Studies that scored less than 6 were judged to be of low quality.

For cross-sectional studies, we used the guidelines for critical appraisal, developed by the National Collaborating Center for Environmental Health [26]. We assessed representativeness of the study participants, methods for ascertaining exposure; comparability of exposure groups (including unexposed) in terms of age, sex, socio-economic status, and HIV risk factors, non-response bias; determination and validation of outcomes; internal validity, and how confounding factors were assessed and addressed. After reviewing the quality of each included study on the basis of these criteria, we assigned a composite quality score of 0–4. Two reviewers (YL and JE) assessed study quality and reached a consensus score for each included study.

### Data extraction

Data from eligible studies were independently abstracted by two reviewers (YL and JE). Differences were resolved by consensus among all reviewers. Studies were stratified by design (cohort, case-control and cross-sectional studies). For cohort studies, the number of subjects in the cohort and the number of incident cases of HIV/AIDS in the IPV-exposed and non-exposed groups were extracted. For case-control studies, information about the size of cases and controls, including the number of cases and controls exposed and unexposed to IPV, were extracted. For cross-sectional studies, data on number of persons in the study groups and number of persons exposed/unexposed to IPV were extracted from the comparison groups. We also extracted data on sample size, age of individuals in the study, data collection methods and study location.

### Data analysis

First, we conducted a systematic review by summarizing, comparing and contrasting the extracted data. Second, we conducted meta-analyses by type of IPV (“physical,” “sexual,” “physical and sexual,” “psychological” and “any type”) and by study design. We calculated pooled odds ratios (ORs) for case-control studies and cross-sectional studies, and relative risks (RRs) for cohort studies, using RevMan software Version 5.0 [27]. Third, we assessed heterogeneity, using the Q test [28] and the *I* ^2^ statistic [29]. Fourth, we assessed sources of heterogeneity by conducting sub-group analyses by (1) region of the study (high-income or low-/middle-income regions of the world), (2) type of instrument used to collect data on IPV. To moderate effect estimates, we analyzed all data using the random effects model irrespective of heterogeneity level. Finally, sensitivity analyses were undertaken to evaluate the stability of the relationship between IPV and HIV infection among women.

## Results

### Description of studies

**Figure 1:**
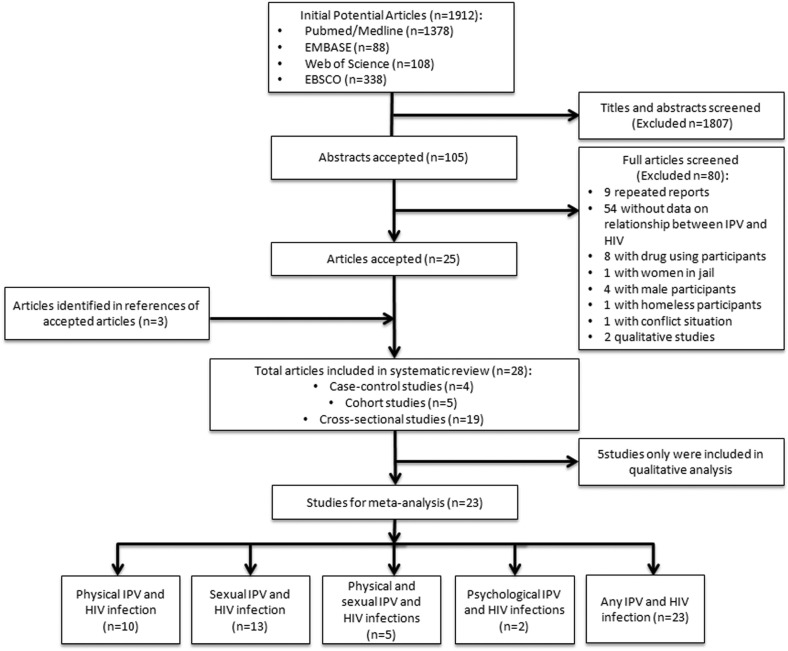
Literature search outputs. This figure describes the full search process and the outputs.

We included 28 eligible studies (19 cross-sectional, five cohort and four case-control) involving 331,468 individuals in 16 countries, the US (eight studies), South Africa (four studies), East Africa (10 studies), India (three studies), Brazil (one study) and multiple low-income countries (two studies) (Figure 1). These studies were summarized in a systematic review [19, 21, 22, 30–54] (Table 1). Only 23 of the 28 studies met the criteria for inclusion in the meta-analysis.

**Table 1:** Characteristics of included studies

Studies	Study design	Country	Sample size	Age group	Violence (period, type)	Data collection instrument	Outcome/results
Dunkle et al. [19]	CS	South Africa	1366	≥16	Lifetime physical, sexual IPV, both physical and sexual IPV	WHO instrument	Any type of IPV was significantly associated with HIV+ [AOR (95% CI): 1.48 (1.15, 1.89)]
Harling et al. [21]	CS	Ten low- to middle-income countries	231,564	15–49	With most recent partners, Physical and sexual IPV, both physical and sexual IPV	Domestic Violence module	IPV was not consistently associated HIV+ among women. Country-specific AOR (95% CI) for physical and sexual IPV ranged from 0.41 (0.12, 1.36) in Haiti to 1.41 (0.26, 7.77) in Mali; pooled AOR (95% CI): 1.05 (0.90, 1.22).
Sareen et al. [22]	CS	USA	13,928	≥20	Last year physical and sexual IPV, both physical and sexual IPV	CTS	Any type of IPV was significantly associated with HIV+ [AOR (95% CI): 3.44 (1.28, 9.22) for any IPV, 8.47 (1.65, 43.57) both physical and sexual IPV]
Speizer et al. [30]	CS	South Africa	3865	1524	Lifetime individual sexual IPV	National household survey questionnaire	Sexual IPV was significantly associated with HIV+ [AOR (95% CI): 1.17 (1.03, 1.32)]
Jewkes et al. [31]	CS	South African	1295	15–26	Lifetime physical/sexual IPV	WHO instrument	Any type of IPV was not significantly associated with HIV+ [AOR (95% CI): 1.16 (0.78, 1.73)]
Were et al. [32]	Follow-up	Seven African countries	1109	≥18	Physical, verbal IPV, both verbal and physical IPV in the past three months	Investigator designed questionnaire	Any type of IPV was not significantly associated with HIV+ [AOR (95% CI): 1.62 (0.59, 4.47)]
Jewkes et al. [33]	Cohort	South African	1099	15–26	Lifetime, physical, sexual IPV in the past three months	WHO instrument	Any type of IPV was significantly associated with HIV+ [adjusted IRR (95% CI):1.51 (1.04, 2.21)]
Kiarie et al. [34]	Baseline data of Cohort	Kenya	2836		Lifetime physical, sexual, psychological, financial IPV	Investigator designed questionnaire	Any type of IPV was significantly associated with HIV+ [adjusted IRR (95% CI):1.2 (0.9, 1.6)]
Burke et al. [35]	CC	USA	611(310 HIV+/301 HIV−)	18–30	Lifetime physical, sexual IPV, both physical and sexual	Investigator designed questionnaire	IPV was not significantly associated with HIV+. Rates of IPV did not differ between HIV− and HIV+ women (66% VS 63% for physical or sexual IPV, 63% VS 62% for any physical, 22% VS 22% for any sexual IPV, 19% VS 20% for both physical and sexual IPV, 45% VS 41% for only physical IPV, 3% VS 4% for only sexual IPV)
Maman et al. [36]	CS	Tanzania	245	≥18	Lifetime physical and sexual IPV	CTS	IPV was significantly associated with HIV+ [AOR (95% CI): 2.42 (1.20, 4.87) for physical violence; 2.39 (1.21, 4.73) for sexual violence; 9.99 (2.67, 37.37) for the younger] (<30)
Quigley et al. [37]	CC	Uganda	133(46 HIV+/87 HIV−)	≥15	Sexual IPV in the past year	Investigator designed questionnaire	Sexual IPV was significantly associated with HIV+ [AOR (95% CI): 7.84 (1.29, 47.86)]
Zablotska et al. [38]	Cohort	Uganda	3422	15–24	Sexual IPV in the past year	Investigator designed questionnaire	Sexual IPV was not significantly associated with HIV infection [AOR (95% CI): 1.23 (0.82, 1.85)]
Barros et al. [39]	CS	Brazil	3193	15–49	Lifetime, physical, sexual, psychological IPV	WHO Instrument	Psychological IPV had no association with HIV[AOR (95% CI): 1.12 (0.7, 1.8)], severe and recurrent any type of IPV was significantly associated with HIV+ [APR (95% CI): 1.91 (1.3, 2.8)]
Wyatt et al. [40]	CC	USA	980(490 HIV+/490 HIV−)	≥18	Lifetime sexual IPV	Investigator designed questionnaire	Sexual IPV was significantly associated with HIV+ (European American: χ_1_^2^=11.72, *p*<.001, African American: χ_1_^2^=7.58, *p=*.006, and Latina: χ_1_^2^=8.85, *p=*.003)
McDonnell et al. [41]	CS	USA	611	>18	Lifetime physical and sexual IPV, both physical and sexual IPV	Investigator designed questionnaire	Physical IPV ≥3 times was significantly associated with HIV infection [AOR (95% CI): 2.15 (1.19–3.86)]
Silverman et al. [42]	CS	Indian	28,139	≥15	Lifetime physical, sexual IPV, both physical and sexual IPV	CTS	Combination of physical and sexual IPV was significantly associated with HIV+ [AOR (95% CI): 3.92 (1.41, 10.94)].
Decker et al. [43]	CS	Indian	20,425	15–49	Lifetime physical and sexual IPV	CTS	Any type of IPV was significantly associated with HIV+ [AOR (95% CI): 7.22 (1.05, 49.88)]
Prabhu et al. [44]	CS	Tanzania	2436	≥18	Lifetime physical and sexual IPV	WHO instrument	Any type of IPV was significantly associated with HIV+ [AOR (95% CI):1.63 (1.01, 2.61)] for the single women
NIMH Group [45]	CS	USA	535	43.41±8.07	Lifetime adult Sexual Abuse (ASA)	The modified Wyatt Sex History Questionnaire	Sexual IPV was significantly associated with HIV+ [OR (95% CI): 1.74 (1.22, 2.48)]
Laughon et al. [46]	CS	USA	445	≥18	Lifetime, past and current physical, sexual IPV	CTS	Any type of IPV was not significantly associated with HIV+. No marked difference in percentage experiencing IPV (current or past) among HIV infected women versus HIV uninfected women (32% VS 37%, 4% VS 9%, 38% VS 33%
Kayibanda et al. [47]	CS	Rwanda	2715	15–49	Lifetime physical, psychological and sexual IPV	CTS	Psychological IPV was significantly associated with HIV+. Women with a score from 3 to 4 on the psychological IPV scale had higher risk for HIV infection [AOR (95% CI): 3.23 (1.30, 8.03)]
Dude [48]	CS	Rwanda	2496	15–49	Lifetime physical, psychological, sexual IPV,	National DHS questionnaire	IPV was significantly associated with HIV+ [OR (95% CI): 4.95 (1.80, 13.6) for emotional abuse; 3.14 (1.16, 8.53) for sexual abuse; 1.98 (1.13, 3.49) for total IPV)
Fonck et al. [49]	CS	Kenya	520	14–49	Lifetime physical IPV	Investigator designed questionnaire	Physical IPV was significantly associated with HIV+ [AOR (95% CI): 1.8 (1.1, 2.8)]
Cohen et al. [50]	CS	USA	1645	≥18	Lifetime and recent physical and sexual IPV	Violence and sexual abuse questionnaire	Any type of IPV was not significantly associated with HIV+ [OR (95% CI): 0.94 (0.73, 1.21)]
Van der Straten et al. [51]	Cohort	Rwanda	921	18–35	Physical and sexual IPV in the past year	KAP survey questionnaire	Sexual IPV was significantly associated with HIV+ [AOR (95% CI): 1.89 (1.2, 2.96)]
Jones et al. [52]	CC	USA	137(53HIV+/84 HIV−)	18–45	Physical and verbal IPV in the past year	CTS	IPV was significantly associated with HIV+. HIV infected women reported higher verbal and physical IPV before (t113=5:52, *p*<.05, t[113]=4:00, *p*<.05, respectively)
Ghosh et al. [53]	CS	India	22,684	15–49	Lifetime sexual IPV	Investigator designed questionnaire	Sexual IPV was significantly associated with HIV+ [AOR (95% CI): 2.63 (1.53, 4.01)]
Kouyoumdjian et al. [54]	Cohort	Uganda	10,252	15–49	Sexual IPV, physical, verbal or any IPV in Lifetime in the past year	CTS	Any type of IPV was significantly associated with HIV+ [adjusted IRR: 1.55 (95% CI): (1.25–1.94)]

CS, cross-sectional studies; CC, case-control studies; IPV, intimate partner violence; CTS, Conflict Tactics Scale; AOR, adjusted odds ratio; IRR, incidence rate ratio; PR, prevalence ratio; DHS, Demographic and Health Survey; KAP, knowledge, attitude and practice.

Study quality assessment (Supplementary file) showed that all included cohort and case-control studies were of good quality (score of 7 or higher). Seven of the 19 cross-sectional studies [21, 22, [41, 42, 43, 47, 48] had a high-quality score of 4; 10 had a score of 3, due to poor representativeness [19, 30, 49], comparability [31, 34], [44, 45, 46, 50] and/or low-response bias [53]. Two had a low score of 2, due to poor representativeness and comparability of the groups [36, 39]. The following section presents results of the systematic review of all 28 eligible studies.

### Systematic review

#### Instruments used to measure IPV

The included studies used different types of instrument to measure IPV (Table 1). These included the Conflict Tactics Scale (CTS) [55], used by eight studies [22, 36, 42, 43, 46, 47, 52, 54]; the WHO Violence Against Women Instrument [56], used by five studies [19, 21, 33, 39, 44], and investigator-designed questionnaire, used by 10 studies [33, 34, 35, 37, 38, 40, 41, 49, 50, 51]. Other national survey tools (Wyatt Sex History Questionnaire and the Demography and Health Survey's Domestic Violence Module) were also used [21, 30, 45, 48]. The most frequently reported IPV types were physical and sexual or their combination.

#### Impact of IPV on HIV infection among women

*All studies* Of the 28 studies included in the systematic review, 20 demonstrated significant association of IPV with HIV infection among women (Table 1]. Eight of 13 studies that investigated the relationship between any type of IPV and HIV infection among women demonstrated significant association. Six of the seven studies that investigated the relationship between sexual IPV with HIV infection among women revealed a significant association. Studies that investigated the relationship between physical IPV, psychological IPV, and combination of physical and sexual IPV with HIV infection among women also demonstrated significant association.

*By type of study design*Three of the five included cohort studies [33, 51, 54] demonstrated significant associations between IPV and HIV infection among women (Table 1). Similarly, three of the four case-control studies [37, 40, 52] observed significant associations between IPV and HIV infection among women. Of the 19 cross-sectional studies reviewed, 14 demonstrated significant associations of IPV with HIV infection among women.

### Meta-analysis

This section presents results of the meta-analysis component of the review. Twenty-three studies involving 308,410 individuals met the criteria for inclusion in the meta-analyses. Meta-analyses were conducted by type of IPV and by study design.

#### Cohort studies

**Figure 2:**
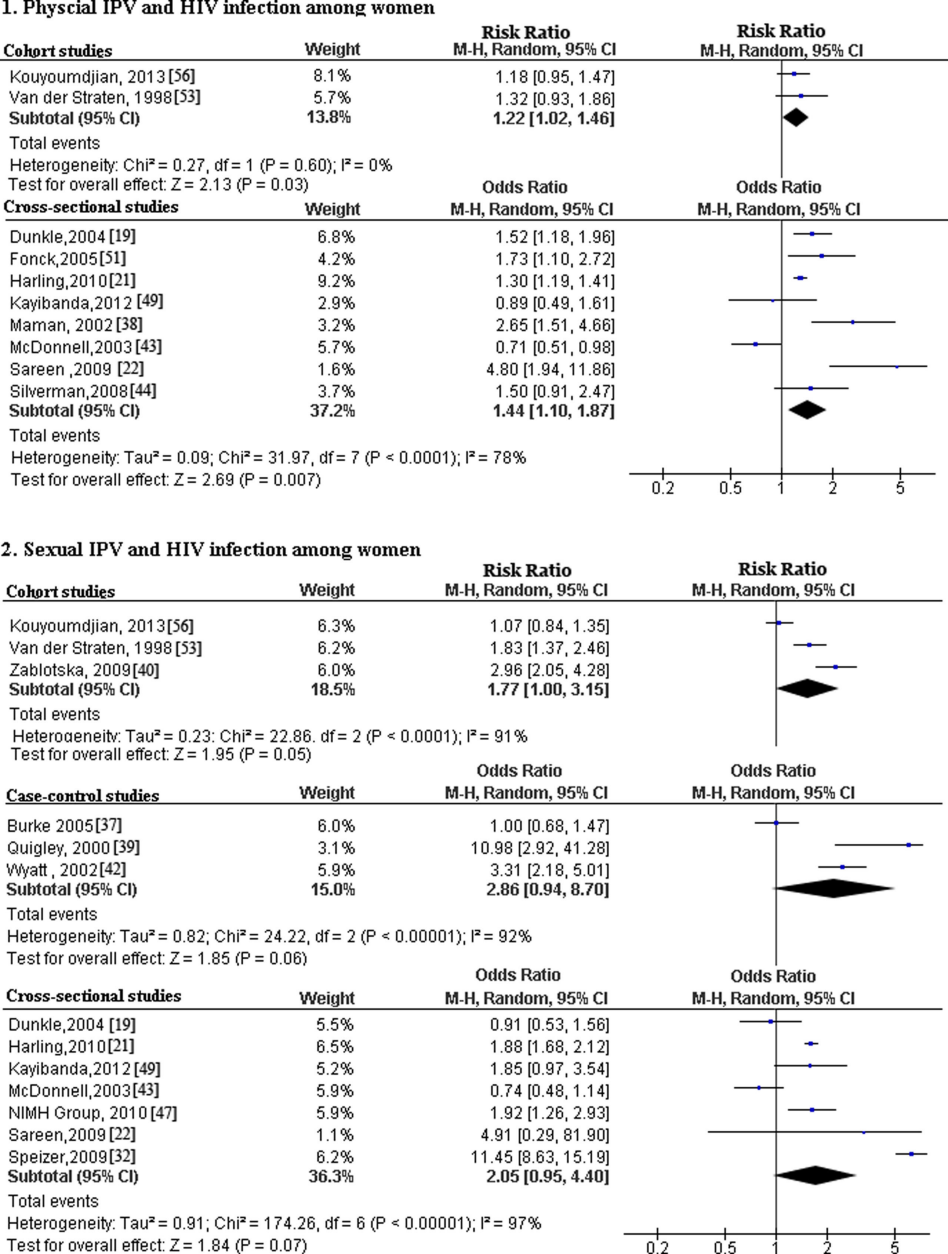
Forest plots of meta-analysis of association of physical IPV and sexual IPV on HIV infection among women. This figure shows forest plots for the meta-analysis of the association between physical IPV and sexual IPV and HIV infection among women in all studies.

**Figure 3:**
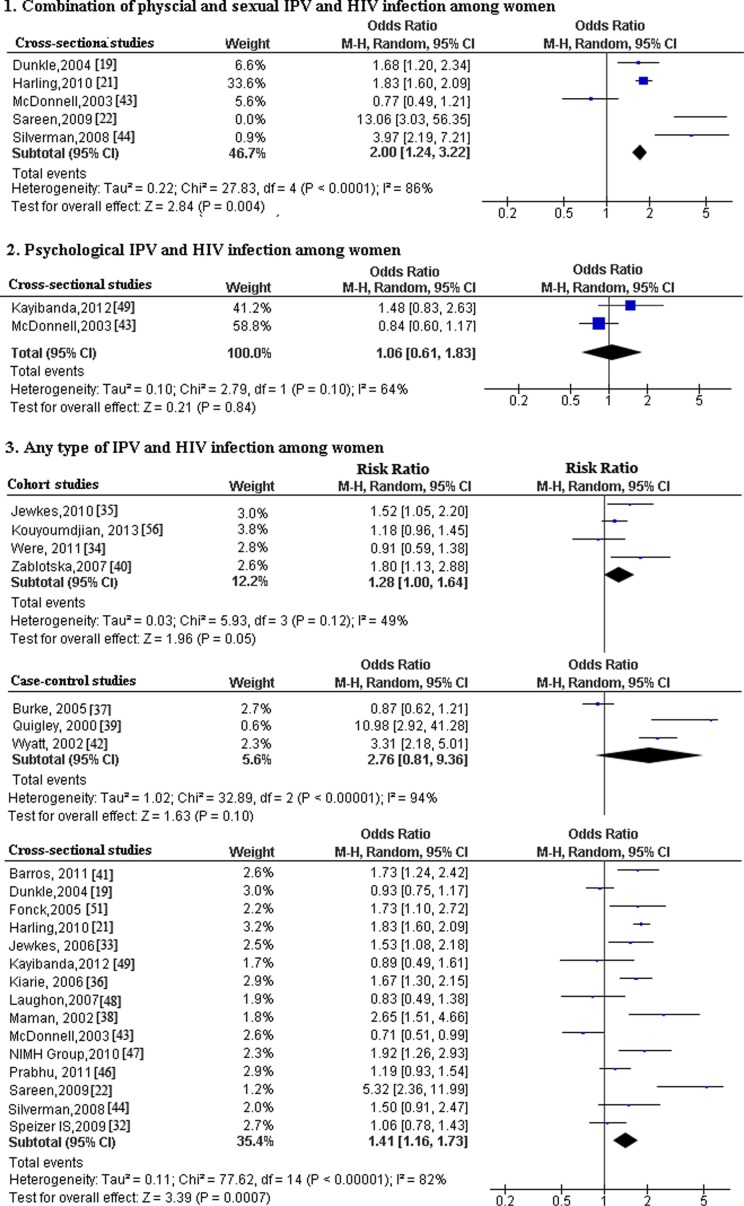
Forest plots of meta-analysis of the association of combination of physical and sexual IPV, psychological IPV and any type of IPV on HIV infection among women. This figure shows forest plots for the meta-analysis of the association of combination of physical and sexual IPV, psychological IPV and any type of IPV (physical, sexual, combination of physical and sexual, and psychological) **and HIV infection among women in all studies**.

While tests indicated that cohort studies on physical IPV, and any type of IPV had no heterogeneity (*I* ^2^=0, *p*=0.6 and *I* ^2^=49%, *p*=0.12, respectively), tests for cohort studies on sexual IPV demonstrated significant heterogeneity (*I* ^2^=91% and *p*<0.00001). Pooled results of cohort studies indicated that physical IPV [pooled OR (95% CI): 1.22 (1.02, 1.46)], sexual IPV [pooled OR (95% CI): 1.77 (1.00, 3.15)] and any type of IPV [pooled OR (95% CI): 1.28 (1.00, 1.64)] were significantly associated with HIV infection among women (Figures 2 and 3).

#### Case-control studies

Marked heterogeneity was indicated for the case-control studies included in meta-analyses of the association of sexual IPV and any type of IPV with HIV infection among women (*I* ^2^>50% and *p*<0.1). Pooled results of case-control studies indicated that sexual IPV [pooled OR (95% CI): 2.86 (0.94, 8.70)], and any type of IPV [pooled OR (95% CI): 2.76 (0.81, 9.36)] were not significantly associated with HIV infection among women (Figures 2 and 3).

#### Cross-sectional studies

Analysis of cross-sectional studies on physical IPV, sexual IPV, combination of physical and sexual IPV, and any type of IPV demonstrated significant heterogeneity (*I* ^2^>50% and *p*<0.1) (Figures 2 and 3), but tests for cross-sectional studies on psychological IPV revealed a lack of heterogeneity among the studies (*I*^2^=64 and *p*=0.10). Pooled results of cross-sectional studies indicated that physical IPV [pooled OR (95% CI): 1.44 (1.10, 1.87)], combination of physical and sexual IPV [pooled OR (95% CI): 2.00 (1.24, 3.22)], and any type IPV [pooled OR (95% CI): 41 (1.16, 1.73)] were significantly associated with HIV infection among women (Figures 2 and 3).

### Subgroup analysis

We explored sources of heterogeneity by conducting sub-group analysis. Based on available data, we conducted sub-group analysis by study region and type of instrument used to collect data on IPV for only the 14 cross-sectional studies (Supplementary file).

#### By study regions

When studies from the United States (high-income country) were excluded from the analysis, we found that for physical IPV, studies in middle-to-low countries had low heterogeneity (*I*^2^=52% and *p*=0.07). Pooled results of the non-heterogeneous studies in middle-to-low countries demonstrated significant association between physical IPV and infection in women [pooled OR (95% CI): 1.47 (1.21, 1.78)].

#### By data collection instruments

We analyzed data by whether studies collected data using the WHO Instrument/DHS Domestic Violence Module [55], the CTS [54], or Investigator Designed Questionnaire. For combination of physical and sexual IPV, two studies [22, 42] that collected data with CTS had low heterogeneity (*I*^2^=56% and *p*=0.13). Pooled results indicated that the combination of physical and sexual IPV was significantly associated with HIV infection among women [pooled OR (95% CI): 5.97 (1.94, 18.41)].

### Sensitivity analysis

We conducted sensitivity analysis to assess impact of studies of low quality [36, 39] by excluding them from pooled analysis of all studies (impact of any form of IPV on HIV infection). Results showed that any form of IPV remained significantly associated with HIV infection [pooled OR (95% CI): 1.32 (1.14, 1.53)]. Similarly, exclusion of the large study of 10 developing countries by Harling et al. [21] did not negatively affect the significance level of the results of all studies for any form of IPV and HIV infection [pooled OR (95% CI): 1.59 (1.16, 2.20)], or for the 14 cross-sectional studies [pooled OR (95% CI): 1.71 (1.11–2.63)].

## Discussion

In discussing the results of the review, it is important to note that the focus of this review is different from the focus of two currently available reviews on the association of IPV with HIV infection in women [23, 57]. The review by Meyer et al. [23] and another by Stockman et al. [57] focused on high-risk women (alcohol abusers, and women who experienced coerced/forced first sexual intercourse as young girls) who do not represent the general population of women globally. Meyer et al. [23] reviewed 45 studies that assessed the impact of substance abuse and violence on HIV-associated risk behaviours and bidirectional relationships between violence and HIV status. They found no difference in experience of IPV between HIV infected and uninfected women in the context of substance abuse. Stockman et al. [57] examined the impact of coerced/forced first sexual intercourse on HIV risk among women in low- and middle-income countries. They found that coerced/forced sexual initiation was associated with HIV/STIs, multiple and high-risk sex partners, and non-use of condom. Reviews by Maman et al. [58] and Campbell et al. [24] did not assess the association of IPV with HIV infection, but provided a framework for understanding the intersections between the two. Thus, there remains a paucity of consistent evidence of the association of IPV with HIV among the general population of women.

To summarize evidence regarding the association of IPV and HIV infection among women, we identified 28 studies involving 331,468 individuals in 16 countries. Pooled analyses of data indicated that physical violence, sexual violence, a combination of physical and sexual violence, and any type of IPV (physical, sexual, or psychological) were associated with HIV infection in women. In particular, results from high-quality cohort studies with no heterogeneity included in this review demonstrated strong evidence of an association between physical and any type of IPV and HIV infection among women. Cross-sectional studies indicated that physical IPV, combination of physical and sexual IPV, and any type IPV were significantly associated with HIV infection among women. Although case-control studies did not indicated significant associations between sexual IPV and any type of IPV and HIV infection among women, pooled results were suggestive of a potential relationship.

As findings of the review demonstrated, the most frequently studied forms of IPV were sexual and physical IPV or their combination. A study by van der Straten et al. [51] demonstrated a relationship between sexual coercion and physical violence, whereby women who reported partners’ insistence to have sex with them against their will were more likely to also report being beaten (OR 1.95; 95% CI] 1.4–2.7). Sexual violence was reported more often as an independent or conjunct risk factor for HIV infection among woman than any other form of IPV.

Sub-group analysis indicated that study region and instruments used to collect data on IPV were sources of heterogeneity. It was notable that IPV was more likely to be significantly associated with HIV infection among women in low- and middle-income countries than in a high-income country. This result underscores the complex factors that underpin HIV infection among women in low- and middle-income countries. For example, the higher prevalence rates of HIV in the low- and middle-income countries could have contributed to the association of IPV and HIV in these countries since the association of IPV and HIV transmission will be weaker in countries where the base prevalence rate of HIV is lower than in countries where the base prevalence rates are higher. Similarly, unlike in many high-income countries, studies in sub-Saharan Africa [59] show that the acceptance of wife beating for transgressing certain gender roles is widespread. Studies conducted in South Africa, Uganda and India [60–62] found that IPV was considered a normal part of marital relationships and was justified by 41–90% of female respondents in at least one situation. Due to cultural factors and resource constraints, most women who experience IPV in low- and middle-income countries do not seek care and support for their experience, and this may increase their risk for HIV infection [63]. A study of IPV and HIV sero-discordance in Uganda [64] found that none of the women reported their experience of IPV to law enforcement authorities, and most did not seek care. In many low- and middle-income countries, the belief that a woman is the property of the husband and the failure of authorities to treat sexual violence as a criminal offence discourages reporting of sexual violence and makes leaving abusive relationships difficult, thereby exacerbating the abuse [65] and heightening the risk for HIV infection.

An important theme that was evident in our review and the general literature was the need to harmonize definitions and tools for measuring IPV. Stockman et al. [12] found that most studies that used behaviourally specific terms for assessing sexual IPV found strong associations between sexual IPV and HIV risk behaviours, while studies that used less specific definitions often did not find these marked associations. The authors emphasized the need for future efforts to integrate behaviourally specific terms in the assessment of the prevalence of sexual IPV and its association with HIV risk.

### Limitations

This review included cross-sectional, case-control and cohort studies, which have some inherent limitations. For example, cross-sectional studies measure exposure and disease status simultaneously. Thus, it is difficult to determine the direction of the observed associations. This is particularly true for IPV and HIV infection. Analyses of the pathways of transmission of HIV as a result of IPV suggest that it is the partner who is perpetrating the IPV that puts the woman at risk for HIV transmission. However, most studies do not consider the timing of the woman's relationship with a partner who perpetrates IPV, and thus, cannot demonstrate temporal association with HIV infection. In fact, while IPV is hypothesized to increase a woman's risk for HIV infection, studies have shown that HIV infection can also lead to, or exacerbate existing violence in relationships [21, 30, 44, 47, 51, 66]. Similarly, studies included in this review lacked data on dose-effect relationships between IPV and HIV infection. Equally, data on the length of exposure to IPV varied considerably among the studies. Although most studies collected lifetime IPV data, some collected data on IPV in the past year [21, 37, 38, 51, 52], while others collected data on IPV in the past three months [32]. Finally, IPV is a very sensitive topic, and victims are often ashamed to openly talk about it [19, 31, 41]. Thus, it is possible that the studies may have grossly under-reported the prevalence of IPV due to negative social desirability bias [19, 38].

It is notable that after controlling for heterogeneity and study quality, pooled results still demonstrated an association between IPV and HIV infection among women. Similarly, this review included studies conducted in diverse cultural settings, involving over 300,000 individuals. We followed standard systematic review methods [27] and the Preferred Reporting Items for Systematic Reviews and Meta-Analyses, PRISMA. We sought to moderate effect estimates by conducting analysis of all data using the random effects model. “The random-effects model incorporates the differences between studies in the calculations and (usually) increases the width of the confidence interval around the pooled estimate of effect, thus giving a more conservative estimate of effect” [67]. Thus, notwithstanding the afore-mentioned limitations, the findings provide useful insight into the global debate on the association between IPV and HIV infection among women.

### Implications for research

To further elucidate the strength of the association between IPV and HIV infection among women, there is a need for high-quality follow-up studies conducted in different geographical regions of the world, and among individuals of diverse racial/cultural backgrounds and varying levels of HIV risks. Studies that assess association between frequency, duration, intensity, severity of IPV and HIV infection can strengthen the IPV–HIV hypothesis.

### Implications for practice and policy

Several studies have established that the fear of IPV is a reason for non-disclosure of HIV positive status by women [68]. Similarly, fear of IPV has been shown to be a factor in women's non-adherence to HIV treatment [65, 69]. Thus, understanding the relationship between IPV and HIV/AIDS among women, and assessing gender-based violence in the context of HIV prevention is important. With regard to prevention of IPV, Dunkle and Decker [16] have provided a summary of individual and population level interventions that can be effective. For example, survivors of IPV should be supported to seek HIV prevention and treatment. Initiatives that address HIV/AIDS should include the assessment of vulnerability to IPV and provide appropriate counselling and referral. A number of important tools are available for assessing women's risk for IPV [70, 71]. Similarly, appropriate counselling and case-management protocols are available for use by professionals working in the field of HIV/AIDS prevention and treatment [70]. At the population level, interventions that address the underpinning societal gender norms that perpetuate IPV against women are needed.
